# Effects of laser-assisted cosmetic smile lift gingivectomy on postoperative bleeding and pain in fixed orthodontic patients: a controlled clinical trial

**DOI:** 10.1186/s40510-014-0066-5

**Published:** 2014-12-09

**Authors:** Farhad Sobouti, Vahid Rakhshan, Nasim Chiniforush, Maziar Khatami

**Affiliations:** Orthodontic Department, Dental Faculty, Mazandaran University of Medical Sciences, Sari, Iran; Iranian Tissue Bank and Research Center, Tehran University of Medical Sciences, Tehran, Iran; Department of Dental Anatomy and Morphology, Dental Branch, Islamic Azad University, Tehran, Iran; Laser Research Center of Dentistry, Dental Research Institute, Tehran University of Medical Sciences, Tehran, Iran; Department Of Periodontics, Dental Faculty, Mazandaran University of Medical Sciences, Sari, Iran

**Keywords:** Cosmetic smile lift, Gingivectomy, Diode laser, Orthodontics, Periodontics

## Abstract

**Background and objective:**

Diode lasers are becoming popular in gingival treatment following orthodontic treatments. Despite their merit and clinical implications, postoperative pain and bleeding after surgery with diode lasers are not assessed except in few controversial studies.

**Method:**

This controlled clinical trial was conducted on 30 healthy orthodontic patients aged 17–29 years, needing esthetic-only gingivectomy in the anterior maxilla. The patients were randomly divided into two groups of 15 each: experimental (laser-assisted surgery) and control (traditional surgery using scalpels). The bleeding rate following the surgery was assessed using the bleeding criteria established by the World Health Organization. The postsurgical pain level was recorded using visual analog scales immediately after the surgery and in patients who consumed analgesics, also 2 h after the analgesic consumption. The data were analyzed using the independent-samples *t*, Mann-Whitney U, and chi-square tests (α = 0.05).

**Results:**

The average bleeding rates were 1.15 and 0.36 in the conventional and laser groups, respectively (Mann-Whitney U *P* < 0.05). Experimental patients had no postsurgical pain (VAS1 and VAS2 = 0). In the control group, the average VAS1 pain was 5.2 out of 10. The difference between VAS1 values in the control/experimental groups was significant (Mann-Whitney U *P* < 0.001).

**Conclusion:**

940-nm diode laser seems promising in reducing postoperative bleeding and pain of patients needing cosmetic smile lift surgeries.

## Background

With an ever-increasing number of adults seeking orthodontic treatment, the improvement of patients’ esthetics has become one of the main goals of orthodontics [1-5]. The gingival esthetics plays a major role in this regard [6]. Disproportionate dentogingival relationships might negatively affect the outcome of treatment, even if the teeth are perfectly aligned [7,8].

Orthodontic treatment might affect gingival health [9-11]. In certain cases, the gingival margin needs recontouring by means of gingivectomy [12]. However, the costs and postsurgical pain of this treatment might discourage patients, unless in severe cases [7,13]. Pain is one of the most important and common postoperative complications, which can discourage patients from seeking treatment; and its proper control might leave a good impression on the patient regarding the quality of surgery [14-20].

Lasers have been useful in various fields, including orthodontics [21]. With the introduction of soft tissue diode lasers, which might be economic and less painful than conventional methods, the gingivectomy treatment became a routine part of orthodontic treatment. Diode lasers might provide proper hemostasis, reduce the infection risk, and prevent damage to the teeth and bone because of their effect range which is limited to soft tissue [7]. They also might improve esthetics while improving soft tissue healing [3,6,22]. Edema, less swelling, and faster healing are the advantages of laser usage in soft tissue management [23,24].

Gingivectomy can be performed by different means such as scalpels, electrosurgery, chemosurgery, and laser [25]. The conventional surgery performed by a small scalpel has been considered the most common method [25-27]. However, the advent of diode lasers highly absorbable by melanin and hemoglobin allows soft-tissue manipulations such as gingival recontouring, operculectomy, or frenectomy accompanied by improved epithelization and wound healing [22,28,29]. Lasers can incise the soft tissue to a depth of 2 to 6 mm [25]. The localized heat causes coagulation, protein denaturization, drying, vaporization, and carbonization at the site of the energy absorption. This might seal blood vessels and inhibit pain receptors at the incision location [27,28]. Therefore, using diode lasers might be advantageous because of better control, potentially lower pain and inflammation, and improved wound healing [22,27,30,31]. Despite these potential advantages, there is only one study comparing traditional method of surgery versus diode laser-assisted surgery in orthodontic setups, which did not enroll a uniform sample of surgeries [27].

In view of the lack of any studies comparing diode laser with scalpel in gingivectomies, this study was conducted. Its objective was to evaluate comparatively the effects of 940-nm diode laser on postoperative bleeding and pain, in orthodontic patients needing cosmetic smile lift gingivectomy. The null hypotheses were that surgery using diode laser versus scalpel would result in a similar level of postoperative bleeding and pain.

## Methods

This controlled clinical trial was conducted on 30 patients undergoing fixed orthodontic treatment during 2012 to 2014. The sample size was predetermined as similar to the previous studies’ sample sizes [25,27,32]. The protocol ethics were approved by the ethics committee of Mazandaran University of Medical Sciences. All patients were aware of their presence in this study, signed the consent form, and could leave at any time. Patients were selected about 1 month before the completion of their orthodontic treatment. The candidate patients were instructed to maintain a proper level of oral hygiene in order to keep their gingivae healthy.

The exclusion criteria comprised non-orthodontic patients, orthodontic patients with poor oral hygiene, orthodontic patients needing ortho-surgical treatment for esthetic purposes, patients having trismus and limited mouth opening, or those with a history of systemic diseases or any kind of disorders that could affect bleeding as well as pain perception directly or through the taken medications.

The inclusion criteria were patients with no/minimum gingival inflammation/pathology but needing esthetic-only gingivectomy (cosmetic smile lift) in bilateral upper incisors and canines after orthodontic treatment. The included patients needed to be skeletally normal and not needing esthetic skeletal surgeries or any underlying bone removals. Their need for esthetic post-treatment gingivectomy would be determined by an orthodontist and a periodontist, based on micro-esthetic criteria for assessing the alveolar bone height.

### Surgery

The orthodontic treatments were performed by an orthodontist at a private clinic in the Sari city. After finishing the treatment, patients who had minimum gingival inflammation were again asked and taught to improve their oral hygiene and plaque control. Two weeks later, they were assessed by the periodontist and orthodontist. They were enrolled in the study if they had no/minimum gingival inflammation. The surgeries were carried out by a periodontist at a private dental center. The patients were randomly divided into two groups of control (15 subjects who received the conventional surgery using scalpels) and experimental (15 subjects who underwent laser gingivectomy). The randomization was done by the orthodontist and periodontist together, according to the order of the approved patients: the approved patients with odd numbers would be sequentially assigned to the conventional group and those with even numbers would be assigned to the laser group.

In the laser group, the local anesthesia was carried out by the topical application of TAC 20 gel (20% lidocaine, 4% articaine, 2% phenylephrine) to the area. Immediately after the beginning of the operation and also during the surgery, patients would be asked about their sensed pain and discomfort. If they sensed any pains, they would receive infiltration injection of 2% lidocaine plus 1:100,000 epinephrine, upon their request until the surgery was performed under complete local anesthesia. In the laser group, no patient asked for extra anesthesia.

In the control (conventional surgery) group, topical TAC 20 gel was applied similar to the laser group. It did not suffice and the patients expressed pain. They received the infiltration injection of 2% lidocaine (1:100,000 adrenaline). The surgeon asked the patient repeatedly regarding any perceived pain or discomfort, in order to make sure that the operation was carried out under absolute anesthetic conditions.

The extent of soft tissue removal in each patient was determined by the periodontist and the orthodontist together. The surgeon used a scalpel (carbon, No. 15 C) in the control group to trim and form the gingival margin. In the laser group, patients were treated for 30 s per tooth by a 940-nm diode laser (diode Epic, BioLase, USA) with a 400-μm fiber at 0.9-W power. During the gingivectomy, the laser tip was held vertically over the gingival margin. By means of a continuous laser beam, the gingival tissue was removed and formed. At the end of the surgery, about a 1-mm gingival sulcus depth remained [23,26].

After achieving the ideal gingival contour and a proper height of the clinical crown, the surgery region was cleansed with a cotton roll or microbrush soaked in 3% hydrogen peroxide. When needed (determined by the periodontist), the area was sutured with 3-0 stitches.

### Bleeding assessment

Postsurgical bleeding was determined in both groups according to the WHO bleeding criteria: (grade 0) no bleeding, (grade 1) bleeding under the skin and petechial class, (grade 2) mild bleeding, (grade 3) gross bleeding, and (grade 4) mortal bleeding or annoying bleeding [33].

### Pain assessment

The pain felt by the patients was evaluated using a visual analog scale (VAS) which was later converted to 11 ranked scores (0: no pain, 10: intolerable pain) as the initial VAS (VAS1) [20,34]. The included patients would be provided analgesics upon their request (Gelophen 400 mg, as many doses as wanted). The time to start taking analgesic was recorded for patients. About 2 h after taking the analgesic capsule(s), pain levels were investigated by a second VAS (VAS2), which was similarly converted to 11 ranks.

As an additional finding, the difference between VAS1 and VAS2 was calculated as an index of analgesic drug effect [27].

Statistical analyses were performed using the independent-samples *t*-test, chi-square and Mann-Whitney U tests of SPSS 20.0 (IBM, USA). The level of significance was predetermined as 0.05.

## Results

More than 300 fixed orthodontic patients who were supposed to be bracket-debonded in a month were evaluated during a 21-month period in 2012 to 2014 until the predetermined number of patients with no or minimum gingival inflammation but needing esthetic-only gingivectomy were enrolled. The excluded patients mostly needed additional or other treatments. Of the evaluated patients, 40 had had healthy gingivae needing esthetic issues in the anterior maxilla. Of these, 4 needed skeletal or bone manipulations and thus were excluded. Six were dropped out later (and replaced by new patients) because of their unacceptable gingival health at the surgery session (determined by the periodontist). The included patients aged 17 to 29 years old. Of them, 12 were males and 18 were females (Table 1). The average ages of the patients in the laser/experimental groups were not significantly different, according to the *t*-test (*P* = 0.974). The gender distribution was not significantly different between the two groups (chi-square *P* = 0.456).

**Table 1:** **Baseline age (year) and gender of the sample**

**Group**	**Laser**		**Control**		**Total**	
**Sex**	**Male**	**Female**	**Male**	**Female**	**Male**	**Female**
Number	5	10	7	8	12	18
Mean age (year)	20.3	21.9	21.3	21.3	20.9	21.6
Standard deviation	3.4	3.3	3.6	3.4	3.4	3.3
Minimum	17	19	18	18	17	18
Maximum	25	29	28	28	28	29
95% CI upper limit	16.0	19.5	17.9	18.5	18.7	20.0
95% CI lower limit	24.6	24.3	24.6	24.2	23.0	23.3

CI, confidence interval.

No harms were reported other than the pre-specified outcomes of pain-bleeding. None of the experimental patients needed suturing, scalpel incisions, or injecting local anesthesia. Nevertheless, 11 patients in the control group (73.3%) needed suturing. The difference between the frequencies of suturing in both groups was statistically significant according to the chi-square test (*P* < 0.001).

### Bleeding

The average bleeding rate in the control group was 1.15 (out of 4). This was significantly higher (Mann-Whitney U *P* < 0.05) than the bleeding rate observed in the experimental group (0.36 out of 4).

### Infiltration injection of local anesthesia

None of the patients in the laser group requested additional anesthetic doses. However, all the patients in the conventional group asked for extra local anesthesia. The difference between two groups in terms of their request for receiving analgesics was statistically significant (chi-square *P* < 0.001).

### Postoperative pain

The experimental patients had no postsurgical pain (VAS1 = 0). In the control group, the average pain was 5.2 out of 10. The VAS1 difference was significant (Mann-Whitney U *P* < 0.001).

### Analgesic consumption

Of the control and experimental subjects, 14 and none consumed analgesics, respectively. The 14 control subjects received the painkillers after an average of 82 min. The frequency of analgesic consumption in the two groups was as well significantly different (chi-square *P* < 0.001). About 2 h after taking the analgesics, the pain felt by the control subjects decreased to 1.2.

## Discussion

Previous studies have compared the laser with conventional surgery [25,27,32]. However, there are no studies comparing the 940-nm diode laser with the conventional surgery in gingivectomy patients, only. In the current study, the bleeding rate in the laser group was less than that in the control group. This was consistent with other studies on the decreased bleeding after laser-assisted surgery [25,27,32,33]. Laser can incise accurately, has a rather deep penetration, can induce coagulation, and is highly absorbed by hemoglobin. All of these factor might contribute to its appropriate hemostasis [7,22,25,27,28,30-33].

Better coagulation also provides a dry and isolated environment, which allows a better control and less infection rate. This might be associated with reduced postsurgical pain [20,35,36]. The laser-assisted surgery had an astounding effect on pain among our patients. None of the patients treated with laser requested analgesics, while almost all control patients asked to receive painkillers. This was in agreement with other studies that reported a reduced pain level by using lasers [25,27,32]. This might be attributed to the less tissue trauma caused by the laser method [35,37]. The lower need for suturing in the laser group might as well imply this.

This clinical trial was limited by some factors. The subjective quality of pain influenced by interindividual and cultural/demographic variations affects its assessment [20,34,38-44]. Therefore, we used VAS, which is understandable by most patients, has proper sensitivity, and is reliable/reproducible [38-40,44,45]. As another limitation, analgesic consumption is a confounder of pain [20,34,46,47]. Therefore, we recorded the pain also before the analgesic consumption. Another limitation was the lack of blinding. However, the patients were not aware of the potential effects of laser on bleeding and pain. Thus, their responses were less likely biased by their knowledge of their group allocation. Moreover, it seemed impossible to blind the operator or the patient in such a design. The generalizability of this study might be reduced by the uniform sample of fixed orthodontic patients all needing esthetic-only gingivectomies, as this is not the case in everyday orthodontic practice. However, such a uniform sample was necessary to establish a proper level of internal reliability. Moreover, the results of this laser cannot be necessarily generalized to other types or wavelengths of lasers. Future multicenter trials performed by different surgeons with different levels of experience might favor the generalizability. As well, such studies can be benefited from split-mouth designs.

## Conclusion

Within the limitations of this clinical trial, it seems that the use of 940-nm diode laser in gingivectomy surgery of canine-to-canine cosmetic smile lift might reduce postsurgical pain and bleeding compared to the traditional method of surgery using scalpels. Laser-assisted surgery might also reduce the need for suturing and patients’ demand for analgesics.
